# Management of ischemic optic neuropathies

**DOI:** 10.4103/0301-4738.77024

**Published:** 2011

**Authors:** Sohan Singh Hayreh

**Affiliations:** Department of Ophthalmology and Visual Sciences, College of Medicine, University of Iowa, Iowa City, IA, USA

**Keywords:** Anterior ischemic optic neuropathy, arteritic anterior ischemic optic neuropathy, non-arteritic anterior ischemic optic neuropathy, non-arteritic posterior ischemic optic neuropathy, posterior ischemic optic neuropathy

## Abstract

Ischemic optic neuropathies (IONs) consist primarily of two types: anterior ischemic optic neuropathy (AION) and posterior ischemic optic neuropathy (PION). AION comprises arteritic AION (A-AION: due to giant cell arteritis) and non-arteritic AION (NA-AION: due to other causes). PION consists of arteritic PION (A-PION: due to giant cell arteritis), non-arteritic PION (NA-PION: due to other causes), and surgical PION (a complication of several systemic surgical procedures). These five types of ION are distinct clinical entities etiologically, pathogenetically, clinically and from the management point of view. In the management of AION, the first crucial step with patients aged 50 and over is to identify immediately whether it is arteritic or not because A-AION is an ophthalmic emergency and requires urgent treatment with high-dose steroid therapy to prevent any further visual loss in one or both eyes. Patients with NA-AION, when treated with systemic corticosteroid therapy within first 2 weeks of onset, had significantly better visual outcome than untreated ones. Systemic risk factors, particularly nocturnal arterial hypotension, play major roles in the development of NA-AION; management of them is essential in its prevention and management. NA-PION patients, when treated with high-dose systemic steroid therapy during the very early stages of the disease, showed significant improvement in visual acuity and visual fields, compared to untreated eyes. A-PION, like A-AION, requires urgent treatment with high-dose steroid therapy to prevent any further visual loss in one or both eyes. There is no satisfactory treatment for surgical PION, except to take prophylactic measures to prevent its development.

Ischemic optic neuropathies (IONs) constitute a major cause of blindness or seriously impaired vision among the middle-aged and elderly population, although no age is immune. Their pathogeneses, clinical features and management have been controversial, resulting in confusion. This article is based on the cumulative information drawn from my basic, experimental and clinical research on various aspects of IONs since 1955, as well as from the literature on the subject.

## Terminology

**Figure 1:**
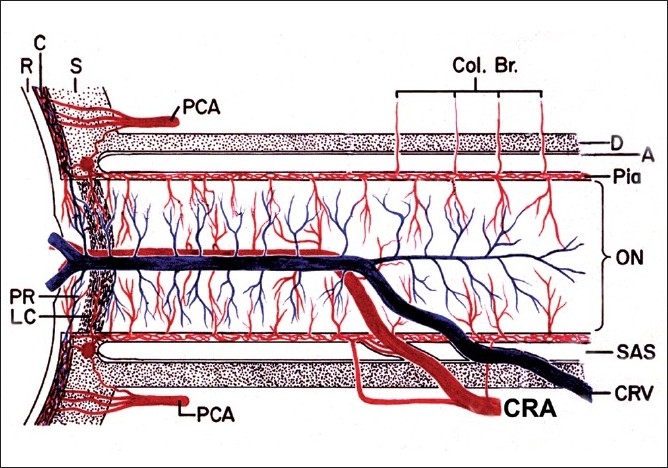
Schematic representation of blood supply of the optic nerve (A = arachnoid; C = choroid; CRA = central retinal artery; Col. Br. = collateral branches; CRV = central retinal vein; D = dura; LC = lamina cribrosa; ON = optic nerve; P = pia; PCA = posterior ciliary artery; PR = prelaminar region; R = retina, S = sclera; SAS = subarachnoid space)

A scientifically valid term is essential in describing a clinical entity. It should reflect true nature of the disease. Before 1974, IONs were described under multiple eponyms.[1] The generic term “ischemic optic neuropathy”, which is widely used, is inadequate, as is evident from the following discussion. On the basis of blood supply, the optic nerve can be divided into two distinct regions: (1) the anterior part of optic nerve head (ONH), which is supplied primarily by the posterior ciliary artery (PCA) circulation and (2) the rest of the optic nerve, which is not supplied by the PCAs but from multiple sources [Fig. 1].[2–7] In the early 1970s, I found from my studies on the blood supply of the optic nerve[2–4] and experimental[8] and clinical[9,10] studies that interference with the PCA circulation resulted in the clinical picture for which I coined the term “anterior ischemic optic neuropathy” (AION).[11] This term represents the exact site and ischemic nature of the lesion in the optic nerve. Later, in 1981, I first described the clinical entity “posterior ischemic optic neuropathy” (PION)[12] which is due to ischemia of a segment of the posterior part of the optic nerve, not supplied by the PCA [Figs. 1b2].

**Figure 2:**
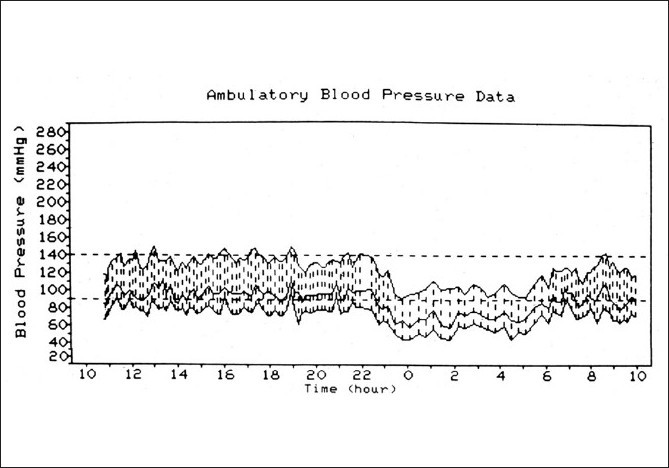
Ambulatory blood pressure monitoring records (based on individual readings) over a 24-hour period, starting from about 11 a.m., in a 58-year-old woman with bilateral NA-AION and on no medication. The blood pressure is perfectly normal during the waking hours but there is marked nocturnal arterial hypotension during sleep[36]

Contrary to the prevalent impression, neither AION nor PION is a single clinical entity; each comprises the following clinical entities with different etiologies, pathogeneses, clinical features and managements.

### Anterior ischemic optic neuropathy

This is of two types as follows.

1. Arteritic AION (A-AION): This is almost invariably due to giant cell arteritis (GCA).
2. Non-arteritic AION (NA-AION): This is due to causes other than GCA.

### Posterior ischemic optic neuropathy

Etiologically, this can be classified into the following three types.

1. Arteritic PION (A-PION): due to GCA.
2. Non-arteritic PION (NA-PION): due to causes other than GCA.
3. Surgical PION: This is attributable to surgical procedures.[13] It has also been called postoperative[14] or perioperative[15] PION. I have used the term “surgical PION”[13] because it is more inclusive.

Thus, the IONs etiologically, pathogenetically, clinically and from the management point of view are actually five distinct clinical entities. AION is far more common than PION. In AION, NA-AION is much more common than A-AION.

There are two essential considerations in the successful management of a disease: (1) Management must have a scientific rationale; any treatment without that, irrespective of how attractive it may seem and how enthusiastic its proponent is, will not be successful in the long run, as was demonstrated by optic nerve sheath decompression in NA-AION (see below). (2) Treatment of a disease must arise from a full understanding of its pathogenesis. In addition to these, a correct diagnosis of the type of ION is crucial to proper management since the treatment of each type of ION is different; this means having a good knowledge of the clinical features of the various types of IONs. Therefore, the subject of management requires a discussion of the following three topics for the various types of IONs: their (1) pathogeneses, (2) clinical features and (3) management. Following is a brief discussion of these three topics, discussed in detail elsewhere.[16]

## Pathogeneses

### Pthogenesis of AION

#### Pathogenesis of A-AION

GCA is the primary cause of A-AION. Other rare causes include other types of vasculitis, such as polyarteritis nodosa, systemic lupus erythematosus, and herpes zoster. GCA is a systemic vasculitis, and it preferentially involves medium-sized and large arteries. In the eye, GCA has a special predilection to involve the PCA, resulting in its thrombotic occlusion. Since the PCA is the main source of blood supply to the ONH [Fig. 1][4,5,7] occlusion of it results in infarction of a segment or the entire ONH, depending upon the area of the ONH supplied by the occluded PCA. This results in development of A-AION, leading to massive visual loss in one or both eyes.

#### Pathogenesis of NA-AION

Pathogenetically, NA-AION is of two types: the most common is caused by transient nonperfusion or hypoperfusion of the ONH circulation; the second, and rarer one, is due to embolism to the arteries/arterioles feeding the ONH. The pathogenesis of NA-AION is discussed at length elsewhere.[16,17] Following is a brief account.

**Figure 3:**
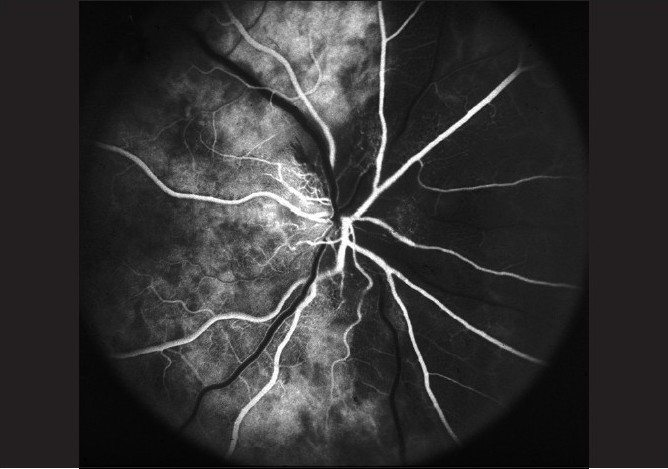
Fluorescein fundus angiogram of right eye with NA-AION (negative temporal artery biopsy for GCA), showing normal filling of the area supplied by the lateral PCA (including the temporal half of optic disc) but no filling of the area supplied by the medial PCA (including the nasal half of optic disc)[36]

- **NA-AION due to transient nonperfusion or hypoperfusion of the ONH circulation:** All the available evidence indicates that *NA-AION is multifactorial* in nature and the risk factors fall into two main categories:
  1. **Predisposing risk factors:** They make the ONH vulnerable to ischemic disorders but do not necessarily produce NA-AION by themselves. These may be systemic or local, in the eye and/or ONH.
    - *Systemic risk factors*: NA-AION patients, compared to the general population, have a significantly higher prevalence of arterial hypertension, diabetes mellitus, nocturnal arterial hypotension, ischemic heart disease and other cardiovascular disorders, hyperlipidemia, atherosclerosis and arteriosclerosis.[17–22] Other reported associated systemic diseases, including sleep apnea,[17,23–25] arterial hypotension from a variety of causes,[26] malignant arterial hypertension,[27] and migraine.
    - *Ocular and ONH risk factors*: NA-AION is significantly associated with absent or small cup in the optic disc,[28,29] the location of the watershed zone of the PCAs in relation to the optic disc,[30] and vascular disorders in the nutrient vessels of the ONH,[5] optic disc drusen and cataract extraction.[31] Other risk factors include markedly raised intraocular pressure (IOP),[17,31] and marked optic disc edema due to any cause.[32]
  2. **Precipitating risk factor(s):** In a person with predisposing risk factors already present, these risk factors act as the final insult, resulting in ischemia of the ONH and NA-AION. In one study, 73% NA-AION patients gave a definite history of discovering the visual loss on waking up in the morning or from a nap, or at the first opportunity in the day to use vision critically.[33] The incidence may actually be much higher than 73% because most of the remaining patients were not certain about the time of the day when they discovered the visual loss. A fall of blood pressure during sleep is almost a universal occurrence. Therefore, nocturnal arterial hypotension is the most important factor in this category [Fig. 2];[33–35] its role is discussed at length elsewhere.[16,17] Furthermore, studies have shown that arterial hypertensives on oral hypotensive therapy have a significant (*P* = 0.004) association between progressive visual field deterioration in NA-AION and nocturnal hypotension.[34,35]
- **NA-AION due to embolism to the arteries/arterioles feeding the ONH:** This is a rare cause of NA-AION. Embolic occlusion of the PCA is seen on fluorescein fundus angiography because the area of the choroid supplied by it does not fill[36] [Fig. 3]. Compared to the hypotensive type of NA-AION, the extent of ONH damage in this type is usually massive, severe, and permanent [similar to that in A-AION (see above)], depending upon the size of the artery involved and the area of the nerve supplied by the occluded artery.

### Pathogenesis of PION

This is discussed at length elsewhere.[13] Briefly, it is as follows.

#### Pathogenesis of A-PION

This is due to GCA when arteritis involves the orbital arteries which supply the posterior part of the optic nerve [Fig. 1]. A-PION occurs much less commonly than A-AION.

#### Pathogenesis of NA-PION

In NA-PION patients, compared to the general population, there is a significantly higher prevalence of arterial hypertension, diabetes mellitus, ischemic heart disease, cerebrovascular disease, carotid artery and peripheral vascular disease and migraine.[13] There are also anecdotal case reports of PION associated with many other diseases.[13,15] Thus, the pathogenesis of NA-PION, like NA-AION,[17] is multifactorial in nature, with a variety of systemic diseases, other vascular risk factors and/or local risk factors predisposing an optic nerve to develop PION; defective autoregulation of the optic nerve may also play a role. Finally, some precipitating risk factor acts as the “last straw” to produce PION. In the vast majority, nocturnal arterial hypotension is the precipitating risk factor [Fig. 2].

#### Pathogenesis of surgical PION

Over the recent years, a large number of surgical PION cases have been reported in the literature, almost invariably associated with prolonged systemic surgical procedures, for a variety of conditions, including spinal and other orthopedic surgical procedures, radical neck dissection, venous graft in extremities, coronary artery bypass, hip surgery, nasal surgery, thoracotomy for hemothorax, penetrating thoracoabdominal injury, cataract surgery, and strabismus surgery.[13] Sadda *et al*,[15] reported 28 patients following a variety of procedures.

The pathogenesis of surgical PION is discussed at length elsewhere.[13] Briefly, it is multifactorial in nature. The main factors include severe and prolonged arterial hypotension (due to prolonged general anesthesia, surgical trauma and massive blood loss), hemodilution from administration of a large amount of intravenous fluids to compensate for the blood loss, orbital and periorbital edema, chemosis and anemia, and rarely even direct orbital compression by prone position. This type of PION usually tends to cause bilateral massive visual loss or even complete blindness, which is usually permanent; therefore, it has great medicolegal importance.

## Clinical Features of Various Types of Ischemic Optic Neuropathies

### Clinical features of Na-Aion

NA-AION is the most common type of ION. It usually has classical symptoms and signs which make it easy to diagnose. The subject is discussed at length elsewhere.[17] Following is a very brief account.

**Figure 4:**
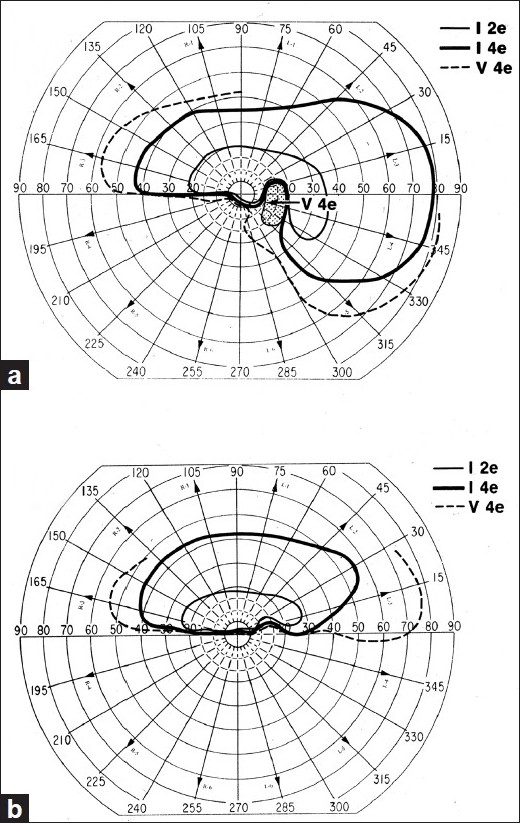
Visual field defects in NA-AION, plotted with Goldmann perimeter (using I-2e, I-4e and V-4e targets). (a) Inferior altitudinal defect with I-2e and inferior nasal defect with I-4e and V-4e; (b) absolute inferior altitudinal defect with I-2e, I-4e and V-4e. The visual acuity in both eyes was 20/20[39]

NA-AION is mostly a disease of the middle-aged and elderly; but contrary to popular belief, it also occurs in young persons, though less commonly. There is a sudden and painless deterioration of vision, usually discovered on waking in the morning.[33] When there is progressive visual loss, the patients again usually notice it on waking in the morning. NA-AION patients often complain of loss of vision toward the nose and, less commonly, altitudinal loss. Later on, photophobia is a common complaint, particularly in bilateral cases. In a study of 500 consecutive NA-AION eyes, when patients were seen within 2 weeks after the onset of visual loss, initial visual acuity was 20/20 in 33%, better than 20/40 in 51%, and 20/200 or worse in 21%.[37,38] This shows that the presence of *normal visual acuity does not rule out NA-AION*. In contrast to this, visual field defects are a universal occurrence. Therefore, *perimetry is the most important and essential visual function test to evaluate the visual loss. A combination of a relative inferior altitudinal defect with absolute inferior nasal defect is the most common pattern in NA-AION*[39] [Fig. 4a]. This contradicts the commonly held belief that inferior altitudinal visual field defect [Fig. 4b] is typical of NA-AION.

**Figure 5:**
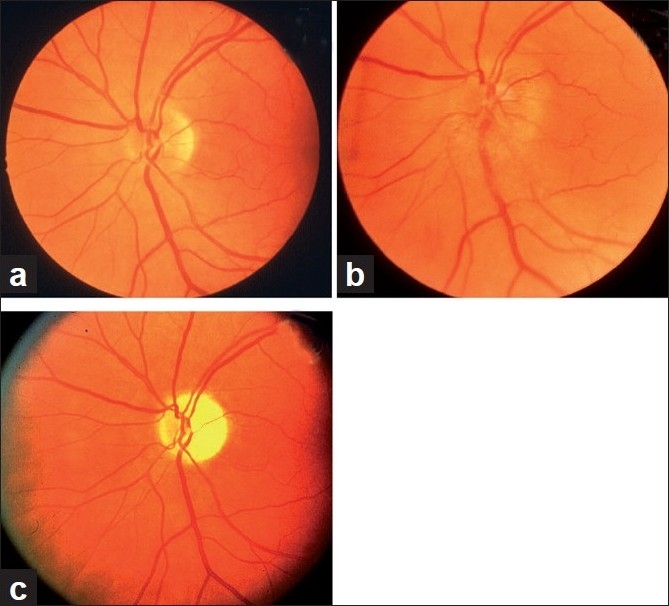
Fundus photographs of left eye of a 53-year-old man: (a) Normal disc before developing NA-AION, (b) with optic disc edema and hyperemia during the active phase of NA-AION, and (c) after resolution of optic disc edema and development of optic disc pallor (more marked in temporal part than nasal part)[16]

**Figure 6:**
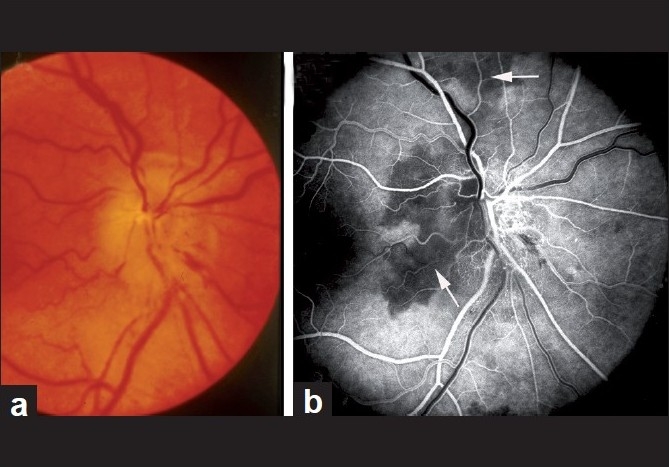
Fundus photograph (a) and fluorescein fundus angiogram (b) of right eye with NA-AION. (a) Optic disc edema, hyperemia and hemorrhages on optic disc; (b) fluorescein fundus angiogram shows non-filling of temporal part of the peripapillary choroid (arrow) and adjacent optic disc and the choroidal watershed zone (arrow)[36]

**Figure 7:**
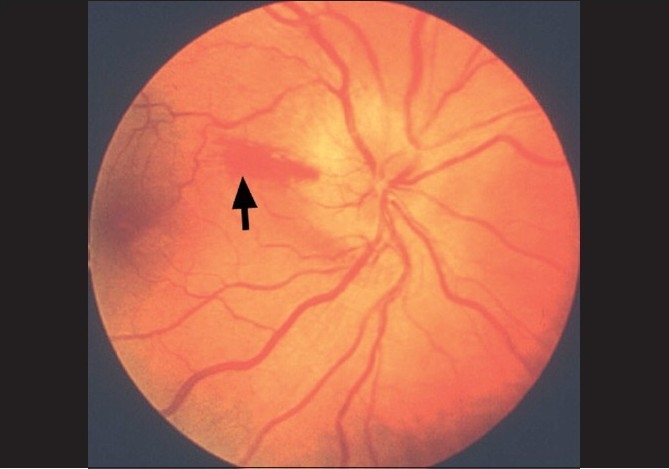
Right fundus photograph showing optic disc edema and hyperemia, with a splinter hemorrhage (arrow) during the acute phase of NA-AION[16]

The main clinical finding on ophthalmic evaluation at the onset of visual loss is optic disc edema [Figs. 5b, 6a, 7][40] which resolves spontaneously in 7.9 (5.8–11.4) weeks, resulting in generalized or sectoral pallor of the optic disc [Fig. 5a–c].[40] There is a characteristic evolutionary pattern of optic disc edema in NA-AION.[40] The presence of a few splinter hemorrhages on the optic disc [Fig. 6a] or immediate peripapillary region [Fig. 7] is common in association with the optic disc edema.

**Figure 8:**
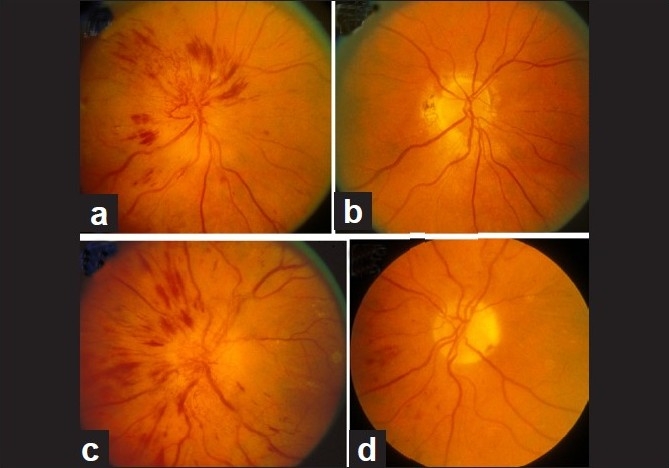
Fundus photographs of the both eyes of a 51-year-old diabetic woman, who developed NA-AION, first in the right eye (a, b) and 8 months later in the left eye (c, d). (a, c) Optic disc edema with marked telangiectatic vessels on the optic disc, multiple punctate peripapillary hemorrhages; (b, d) no edema, no abnormal vessels on the disc, and no peripapillary retinal hemorrhages on resolution[41]

In diabetics, optic disc changes in NA-AION may have some characteristic diagnostic features. During the initial stages, the optic disc edema is usually (but not always) associated with characteristic prominent, dilated and frequently telangiectatic vessels over the disc, and peripapillary retinal hemorrhages that are much more numerous than in non-diabetics[41,42] [Fig. 8a and c]. These findings may easily be mistaken for proliferative diabetic retinopathy associated with optic disc neovascularization. When the optic disc edema resolves spontaneously, these prominent telangiectatic disc vessels and retinal hemorrhages also resolve spontaneously [Fig. 8b and d]. The presence of these characteristic fundus changes in some diabetics with NA-AION has led to a good deal of controversy because it has been thought to be a separate clinical entity that is described under different eponyms, the most common being “diabetic papillopathy”, when in fact it is NA-AION.[42]

**Figure 9:**
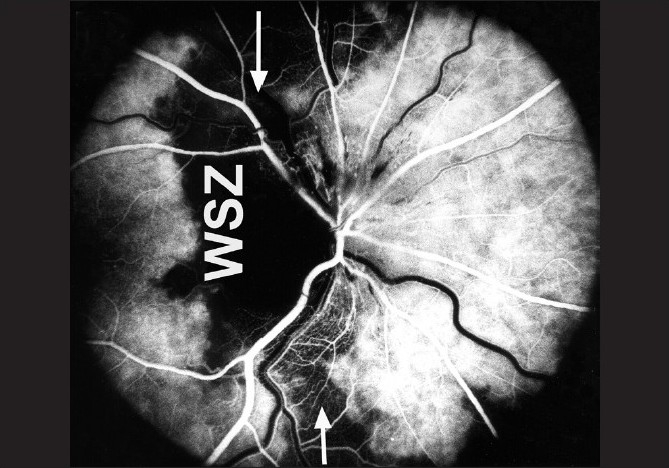
Fluorescein fundus angiogram of right eye with NA-AION, showing location of the watershed zone (arrows, WSZ, vertical dark band) in relation to the optic disc. The watershed zone is passing through the temporal part of the disc and adjacent temporal peripapillary choroid[36]

Fluorescein fundus angiography at the onset of NA-AION *during the very early choroidal arterial phase of dye filling* almost invariably shows filling defect/delay in the prelaminar region and in the peripapillary choroid [Fig. 6b] and/or choroidal watershed zones[36] [Figs. 6b, 9].

### Clinical features of incipient NA-AION

In 1981,[43] I reported that “symptomless optic disc edema precedes the visual loss and may be the earliest sign of AION (NA-AION)”. In 2007, based on a detailed study of a series of 60 eyes with symptomless optic disc edema, I described this as a distinct clinical entity under the name of “*incipient NA-AION. 0”*[44] In this condition, initially there is asymptomatic optic disc edema and no visual loss attributable to NA-AION. Available evidence indicates that it represents the earliest, asymptomatic clinical stage in the evolution of the NA-AION disease process; therefore, it shares most clinical features with classical NA-AION except for the visual loss. In 25%, incipient progressed to classical NA-AION (after a median time of 5.8 weeks). Patients with incipient NA-AION had a greater prevalence of diabetes mellitus than those with classical NA-AION; therefore, this has often been misdiagnosed as “diabetic papillopathy” or “diabetic papillitis”.

### Misconceptions about NA-AION

The subject of NA-AION is, unfortunately, plagued with multiple misconceptions, resulting in controversy and confusion. The subject is discussed elsewhere.[16] Following are the major misconceptions.

1. **Misconception:** NA-AION and cerebral stroke are similar in nature.
  **Reality:** That is not valid because cerebral stroke is a thromboembolic disorder, whereas NA-AION is primarily a hypotensive disorder in the vast majority of cases.[16,17]
2. **Misconception:** Absence of an optic disc cup is the main cause of development of NA-AION.
  **Reality:** That is not correct. An absent or small cup is simply a secondary contributing factor once0 the process of NA-AION has started, and not0 a primary factor.[28,29]
3. **Misconception:** There is no spontaneous visual improvement in NA-AION.
  **Reality:** Two large prospective natural history studies have shown that visual acuity improves spontaneously in 41–43% of the eyes.[37,45]
4. **Misconception:** NA-AION is not seen in young persons.
  **Reality:** Two large studies have disproved this myth.[46,47]
5. **Misconception:** All eyes with NA-AION initially have pale optic disc edema.
  **Reality:** Disc pallor actually starts to develop only 2–3 weeks after the onset of visual loss; before that there is no pale optic disc edema.[40]
6. **Misconception:** Inferior altitudinal defect is the classical diagnostic visual field defect in NA-AION.
  **Reality:** A study of 312 NA-AION eyes showed that inferior nasal field defect is the most common defect.[39]
7. **Misconception:** All eyes with NA-AION have poor visual acuity at onset.
  **Reality:** In a study of 237 eyes seen within 2 weeks of onset, 33% had 20/40 or better visual acuity.[37,38]
8. **Misconception:** Steroid therapy has no role in the management of NA-AION.
  **Reality:** In a study of 696 NA-AION eyes (364 treated versus 332 controls), the treated group showed significantly more visual acuity improvement than the control group (70% versus 41%) (see below).[38]
9. **Misconception:** Smoking is a risk factor for development of NA-AION.
  **Reality:** Two large prospective studies have shown that this is not true.[46,48]
10. **Misconception:** Aspirin reduces the risk of second eye involvement by NA-AION.
  **Reality:** Two large studies have disproved this belief.[48,49]
11. **Misconception:** All patients with NA-AION should be investigated for thrombophilia.
  **Reality:** NA-AION is not a thromboembolic disorder in the vast majority of cases, so this is not necessary.[46,50]
12. **Misconception:** All eyes with NA-AION have visual loss from the start.
  **Reality:** Symptomless optic disc edema is the earliest sign of NA-AION.[43,44]

### Clinical features of A-AION

GCA, which is by far the most common cause of A-AION, is a disease of late middle-aged and elderly persons. In a study of 85 GCA patients with A-AION, mean ± SD age was 76.2 ± 7.0 (range 57–93 years).[51] It is much more common among women than men (71% versus 29%, respectively).[51] There is evidence that GCA is far more common among Caucasians than other races. A few cases have been reported from India.[52] Amaurosis fugax is an important visual symptom of A-AION, present in about one third, and it is an ominous sign of impending visual loss in GCA.[51] Occasionally, diplopia may be present.

Most patients with GCA develop visual loss suddenly without any warning. In one large series of 123 eyes with visual loss due to GCA, initial visual acuity was 20/40 or better in 21%, 20/50–20/100 in 17%, 20/100 to count fingers in 24% and hand motion to no light perception in 38%.[51]

**Figure 10:**
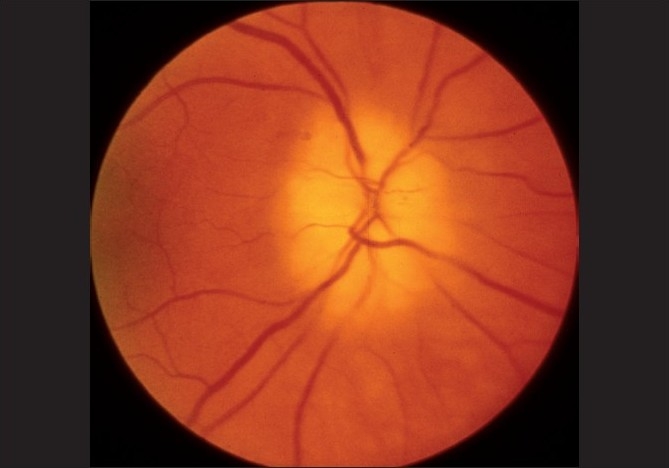
Fundus photograph of right eye with A-AION, showing chalky white optic disc edema during the initial stages[16]

**Figure 11:**
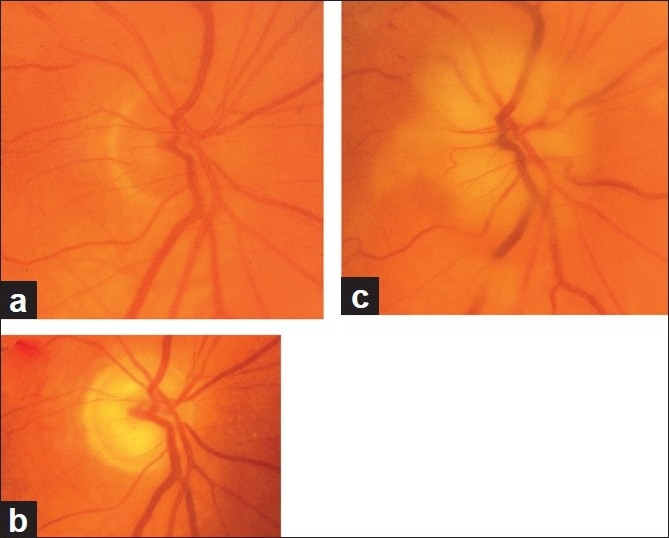
Fundus photographs of right eye with A-AION: (a) Before developing A-AION, (b) 1 week after developing A-AION with chalky white optic disc edema and (c) 4 months later showing optic disc cupping with a cup/disc ratio of 0.8 (note no cup in Fig. 11a before developing A-AION)[16]

**Figure 12:**
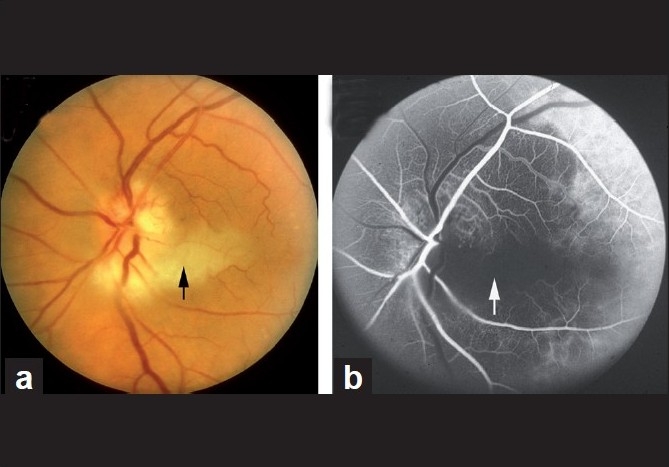
Fundus photograph (a) and fluorescein angiogram (b) of left eye with A-AION associated with cilioretinal artery occlusion. (a) Shows chalky white disc edema temporally and retinal infarct in the region of the occluded cilioretinal artery (arrow). (b) Shows normal filling of the area supplied by the lateral PCA, but no filling of the choroid and entire optic disc supplied by the medial PCA or of the cilioretinal artery (arrow)[1,9–18]

In A-AION, compared to NA-AION, optic disc edema usually has a diagnostic appearance, i.e., a chalky white color[1,9,51] (seen in 69%)[51] [Figs. 10, 11b and 12a]. When optic disc edema resolves, the optic disc in the vast majority shows cupping, indistinguishable from that seen in glaucomatous optic neuropathy, except that there is pallor of the rim[1,9,51] [compare optic disc cup in Fig. 11a with that in Fig. 11c]. By contrast, in NA-AION no such cupping of the optic disc is seen.[1,9,51] Other fundus findings include cotton-wool spots, central retinal artery occlusion, cilioretinal artery occlusion (which is erroneously diagnosed as “branch retinal artery occlusion”), choroidal ischemic lesions and rarely ocular ischemia.[51] Fluorescein fundus angiography performed during the first few days after the onset of A-AION shows the absence of choroidal filling supplied by the occluded PCA [Fig. 12b]; however, later on, with the establishment of collateral circulation, this information may be lost. *A combination of chalky white optic disc edema, retinal infarct in the region of the occluded cilioretinal artery and presence of PCA occlusion on fluorescein angiography* is diagnostic of A-AION [Fig. 12].

### Misconceptions about A-AION and prevention of visual loss in GCA

1. **Misconception:** To diagnose GCA, the patient must have systemic symptoms and signs of GCA.
  **Reality:** This is not valid. A study showed that 21% of GCA patients have no systemic symptoms or signs whatsoever and the only presenting sign is visual loss, i.e., occult GCA.[53]
2. **Misconception:** To diagnose GCA, the patient must have elevated erythrocyte sedimentation rate (ESR).
  **Reality:** This is not true. It has been shown that normal or low ESR (as low as 4–5 mm/hour Westergren) does not rule out GCA.[54]
3. **Misconception:** Steroid therapy can be tapered according to a set regimen.[55]
  **Reality:** On the contrary, there is marked inter-individual variation in the required tapering regimen of steroid therapy and no “one size fits all”.[52]
4. **Misconception:** Steroid therapy can be regulated by using clinical symptoms and signs of GCA.
  **Reality:** This is not valid to prevent visual loss. The only reliable method is to use ESR and C-reactive protein (CRP) as the guideline.[52]
5. **Misconception:** Steroid therapy can be stopped after 1–2 years because the disease burns itself out.
  **Reality:** This is not true at all. The vast majority of patients require life-long steroid therapy to prevent visual loss.[52]

### Clinical features of PION

**Figure 13:**
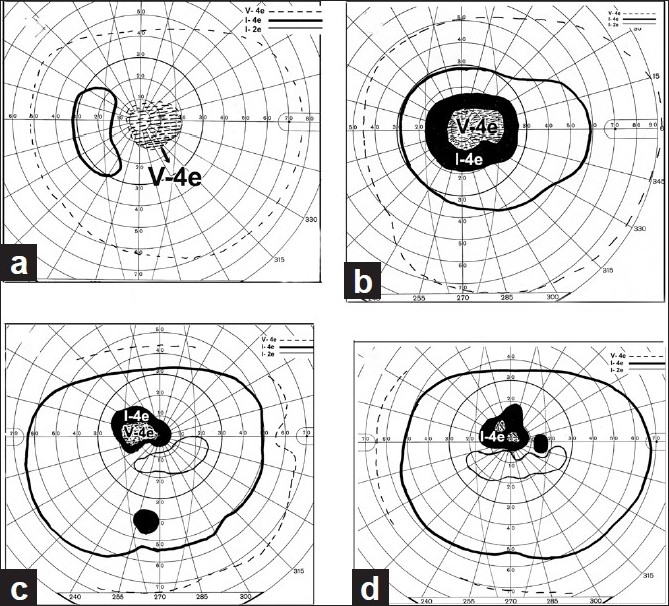
Four visual fields in eyes with NA-PION, showing varying sizes and densities of central scotoma and other field defects, with normal peripheral visual fields[13]

**Figure 14:**
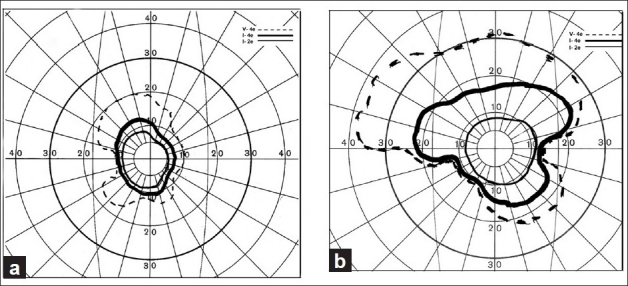
Visual fields of (a) right and (b) left eyes with A-PION, showing markedly constricted central visual fields, with complete loss of peripheral fields in both eyes[13]

PION, like NA-AION, is seen mostly in the middle-aged and elderly population but no age is immune. Clinically, patients with A-PION and NA-PION typically present with acute, painless visual loss in one or both eyes, sometimes discovered upon waking up in the morning. In some eyes, it may initially be progressive. Patients with surgical PION discover visual loss as soon as they are alert postoperatively, which may be several days after the surgery. s0 urgical PION usually tends to cause bilateral massive visual loss or even complete blindness, which is usually permanent. Visual acuity depends upon the type of PION. In one series,[13] it was 20/20–20/75 in 17%, better than 20/40 in 20%, 20/200 or worse in 69% in NA-PION, and 29, 43, 50%, respectively, in A-PION, and in surgical PION often only light perception. The most common visual field defect is central visual loss, alone or in combination with other types of visual field defects [Fig. 13], and much less commonly the reverse pattern, i.e., the central field normal with marked loss of peripheral fields[13] [Fig. 14].

Initially, apart from relative afferent pupillary defect in unilateral PION, the anterior segment, IOP, and optic disc and fundus are normal on ophthalmoscopy and fluorescein fundus angiography. The disc develops pallor, generally within 6–8 weeks, usually more marked in the temporal part.[13]

A combination of the following findings is highly suggestive of PION:

- sudden onset of visual deterioration, with or without deterioration of central visual acuity;
- optic nerve related visual field defects in the involved eye;
- presence of a relative afferent pupillary defect in the involved eye in patients with a perfectly normal fellow eye;
- optic disc and fundus initially normal on ophthalmoscopy and fluorescein fundus angiography;
- no other ocular, orbital or neurological abnormality to explain the visual loss, and
- development of optic disc pallor, usually within 6–8 weeks.

The diagnosis of surgical PION, on the other hand, is relatively straightforward; dramatic visual loss noticed as soon as the patient is alert after a major surgical procedure, with the clinical findings as above. As I stressed in my original paper[12] on PION, *it is a diagnosis of exclusion, and all other possibilities must be excluded before this diagnosis is reached*.

## Management of Ischemic Optic Neuropathies

### Management of AION

*When a patient is diagnosed as having AION, the first crucial step in patients aged 50 and over is to identify immediately whether it is arteritic or non-arteritic*. A-AION is due to GCA. GCA is the most important medical emergency in ophthalmology, rightly characterized by Kearns[56] as “the prime medical emergency in ophthalmology, there being no other disease in which the prevention of blindness depends so much on prompt recognition and early treatment.” Visual loss is preventable if GCA is diagnosed and treated early. Therefore, immediate diagnosis of A-AION and start of high-dose steroid therapy is the key to preventing any further visual loss in the same eye or in both eyes.

### Differentiation of A-AION from NA-AION

This is discussed at length elsewhere.[17,52,57] Collective information provided by the following criteria helps to differentiate the two types of AION reliably.

1. *Systemic symptoms of GCA*: GCA patients usually present with systemic symptoms, including anorexia, weight loss, jaw claudication, headache, scalp tenderness, abnormal temporal artery, neck pain, myalgia, malaise and anemia. A study based on 363 patients who had temporal artery biopsy showed that patients with positive temporal artery biopsy for GCA had a significant association with jaw claudication (odds 9.0 times, *P* < 0.0001), neck pain (odds 3.4 times, *P* = 0.0003), and anorexia (*P* = 0.0005), with no other systemic symptoms showing a significant difference from those with a negative biopsy.[54] Most interestingly, this study showed that 21.2% of patients with visual loss and positive temporal artery biopsy for GCA had *occult GCA*, i.e., no systemic symptoms whatsoever, with visual loss.[53] This is an extremely important clinical entity because there is almost a universal belief that all patients with GCA always have systemic symptoms, which has resulted in missing GCA, with tragic consequence of blindness. Thus, one in five patients with GCA is at risk of going blind without any systemic symptoms of GCA at all.
2. *Visual symptoms*: As discussed above, amaurosis fugax is highly suggestive of A-AION and is extremely rare in NA-AION.
3. *Hematologic abnormalities*: Immediate evaluation of ESR and CRP is vital in all AION patients aged 50 and over. Elevated ESR and CRP, particularly CRP, is helpful in the diagnosis of GCA. Patients with NA-AION do not show any of these abnormalities, except when a patient has some other intercurrent systemic disease. Much less commonly, other hematological abnormalities are seen.[58]
4. *Early massive visual loss*: In our study, initial visual acuity of count fingers to no light perception was seen in 54% with A-AION[51] and in only 14% with NA-AION.[37] This shows that early massive visual loss is extremely suggestive of A-AION. However, the presence of perfectly normal visual acuity does not rule out A-AION (see above).
5. *Chalky white optic disc edema* [Figs. 10, 11b, 12a]: *This is almost diagnostic of A-AION* and is seen in 69% of A-AION eyes. In NA-AION, chalky white optic disc edema occurs only very rarely, with embolic occlusion of the PCA.
6. *A-AION associated with cilioretinal artery occlusion* [Fig. 12]: *This is almost diagnostic of A-AION* because both the ONH and cilioretinal artery derive their blood supply from the PCA, and occlusion of this artery naturally results in both lesions.
7. *Evidence of PCA occlusion on fluorescein fundus angiography* [Fig. 12b]: If angiography is performed during the first few days after the onset of A-AION, the absence of choroidal filling supplied by the occluded PCA *is almost diagnostic of A-AION*. However, later on, with the establishment of collateral circulation, this information may be lost.
8. *Temporal artery biopsy*: This finally establishes the diagnosis. It should be performed in all cases suspected of GCA.

### Management of a-AION

Since it is caused by GCA, management of A-AION is essentially a management of GCA to prevent any further visual loss in the same eye or both eyes. This is a highly controversial subject because practically all the available information is from the rheumatological literature. However, there is a difference in perspective on gca0 between rheumatologists and ophthalmologists, and this has influenced their recommendations on steroid therapy – the regimen advocated by the former primarily concerns managing benign rheumatologic symptoms and signs, whereas the latter confronts the probability of blindness.[52] Moreover, I have found that rheumatologists often tend not to differentiate between polymyalgia rheumatica and GCA in their management. A regimen of steroid therapy, which is adequate to control the rheumatologic symptoms and signs of polymyalgia rheumatica, is often totally inadequate to prevent blindness. With this in view, I did a 27-year prospective study[52] on steroid therapy in GCA, to find a regimen that would prevent visual loss. In the light of information from that study, the following are my guidelines to prevent visual loss.

**Figure 15:**
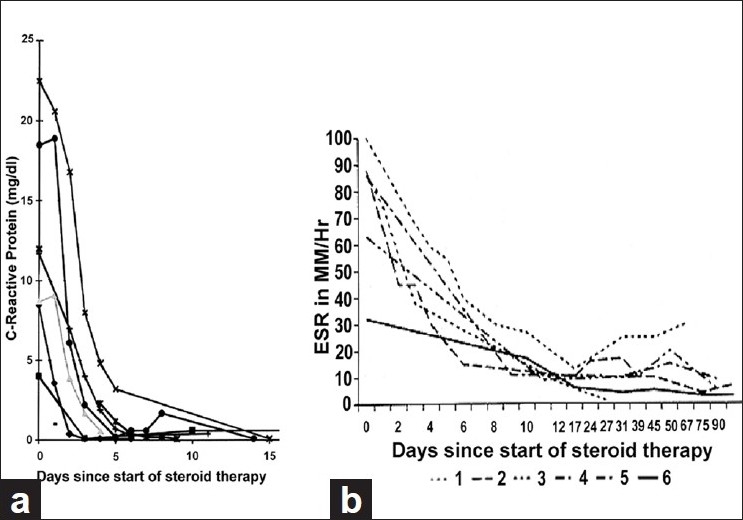
Graphs of (a) CRP levels and (b) ESR of six patients with GCA, showing their initial responses to high-dose steroid therapy[52]

- If there is a reasonable index of suspicion of GCA, as judged from systemic symptoms, high ESR and CRP (particularly high CRP) and sudden visual loss from A-AION, central retinal artery occlusion or cilioretinal artery occlusion, high doses of systemic corticosteroid therapy must be started immediately, as an emergency measure. *The physician should not wait for the result of the temporal artery biopsy* because by the time it is available, the patient may have lost further vision irreversibly, in one or both eyes. Every minute counts; it is unwarranted to take chances of losing vision by starting with a small dose; once vision is lost, a subsequent higher dose will not restore it. In my study, the median starting oral prednisone dose was 80 mg/day, with 40% of patients on ≥100 mg/day.
- High-dose steroid therapy must be maintained until both the ESR and CRP settle down to a stable, low level, which usually takes 2–3 weeks – CRP usually settles much earlier than the ESR [Fig. 15].
- After that, gradual tapering down of steroid therapy should be started. Recently, Salvarani and colleagues[55] stated that 2–4 weeks after the start of initial dose, “the dose can be gradually reduced each week or every 2 weeks by a maximum of 10% of the total daily dose.” My study[52] showed this not to be a safe formula to prevent blindness. To prevent visual loss, *a titration of the steroid dosage with the levels of ESR and CRP is the only safe and reliable method for tapering down and follow-up of steroid therapy*. Using the clinical symptoms and signs of GCA as a guide (often recommended by rheumatologists) is a dangerous practice to prevent blindness.[59] In my study, I found that there is a risk of further visual loss during the first 5 days of the start of high-dose steroid therapy;[60] after that, no one in my study lost any further vision when steroid therapy tapering was guided by the ESR and CRP levels. This showed the effectiveness of steroid therapy in GCA to prevent visual loss, and also that visual loss in GCA is entirely preventable with adequate steroid therapy. In view of this, visual loss due to GCA has become a medicolegal issue. In eyes with visual loss due to GCA, there is only a 4% chance of any visual improvement with steroid therapy.[61]

### Management of NA-AION

This has been a highly controversial subject. A number of treatments have been advocated over the past two decades, principally the following.

**Figure 16:**
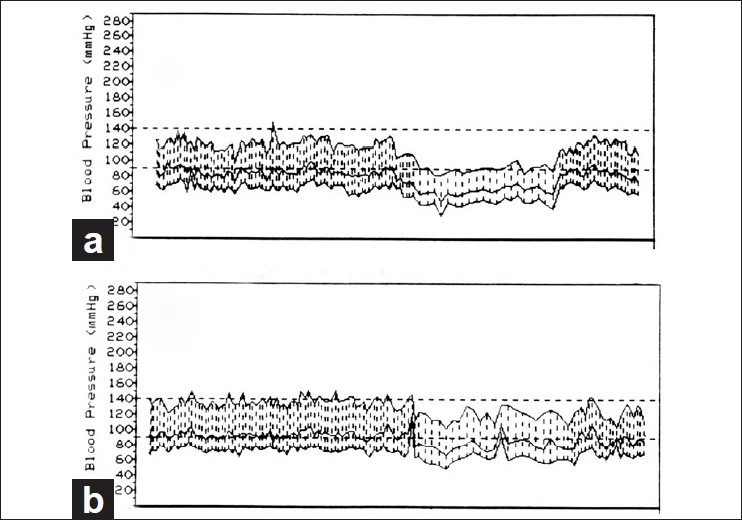
Two 24-hour ambulatory blood pressure monitoring records (based on individual readings), starting at 10 a.m., of a 63-year old woman taking Verapamil hydrochloride for migraine. Both the records show normal blood pressure during the waking hours. (a) Shows that when she was taking Verapamil at bedtime, during sleep there was a marked degree of nocturnal arterial hypotension (blood pressure falling as low as 80/30 mm Hg). (b) Shows markedly less nocturnal hypotension on stopping the bedtime dose of Verapamil (lowest blood pressure 110/50 mm Hg)

1. *Optic nerve sheath decompression*: Sergott *et al*,[62] in 1989 claimed that optic nerve sheath decompression improved visual function in “progressive” NA-AION. The procedure gained worldwide favor not only in “progressive” but also in all types of NA-AION, in spite of a warning that there was no scientific rationale for doing optic nerve sheath decompression in NA-AION and that the procedure could be harmful.[63] A multicenter clinical trial conducted by the National Institutes of Health in the United States subsequently established that this procedure is “not effective” and “not an appropriate treatment for non-arteritic AION” and “may be harmful” because 24% of the eyes with the optic nerve sheath decompression suffered further visual loss as compared to only 12% simply left alone.[45] The multicenter clinical trial study also showed that 42% of cases improved spontaneously in visual acuity, without any procedure.
2. *Aspirin*: A study based on 131 patients claimed that aspirin prevented the development of NA-AION in the fellow eye.[64]
  A much larger study based on 431 patients with unilateral NA-AION revealed no long-term benefit from aspirin in reducing the risk of NA-AION in the fellow eye.[49] Similarly, another large study[48] found no association between regular aspirin use and incidence of new NA-AION in the fellow eye. Botelho *et al*,[65] also found that the use of aspirin does not improve the visual outcome in NA-AION patients. These findings are not surprising since NA-AION is not a thromboembolic disorder, but a hypotensive disorder in the vast majority, and aspirin has no effect on the blood pressure or nocturnal arterial hypotension.
3. *Systemic corticosteroid therapy*: Two small reports almost four decades ago suggested that systemic corticosteroids given during the very early stages of the disease may help to improve the visual function in some patients.[10,66] A recent large, prospective study,[38] based on 696 eyes, comparing the visual outcome in treated (364 eyes) versus untreated control (332 eyes) groups, found that in eyes with initial visual acuity of 20/70 or worse, seen within 2 weeks of onset, there was visual acuity improvement in 70% in the treated group compared to 41% in the untreated group (odds ratio of improvement: 3.39; 95% CI: 1.62, 7.11; *P* = 0.001). Similarly, among those seen within 2 weeks of NA-AION onset with moderate to severe initial visual field defect, there was improvement in 40% of the treated group and 25% of the untreated group (odds ratio: 2.06; 95% CI: 1.24, 3.40; *P* = 0.005). In both treated and untreated groups, the visual acuity and visual fields kept improving for up to about 6 months after the onset of NA-AION, but very little thereafter. A comparison of treated versus untreated groups also showed that optic disc edema resolved significantly (*P* = 0.0006) faster in the treated group.[40] A British review journal called “*Faculty of 1000 Medicine Review – F1000 Medicine – the expert guide to the most important advances in medicine*” concluded that: “This study will be the gold standard recommendation for treatment of non-arteritic ischemic optic neuropathy”.
  Critics have argued that this study was not based on conventional randomization but on “patient randomization”. Therefore, its results are open to criticism. In this criticism, some crucial features in this study have been totally ignored. In any such clinical trial, the most important criterion for assessing the validity of its results is that the baseline parameters of the treated and the untreated groups must be similar. In this study, of the 696 cases, the numbers that volunteered in the “patient choice method” to take steroid therapy and that decided not to take any treatment were not significantly different (364 versus 332). A comparison of demographic, clinical and other characteristics of the cohort that volunteered to take steroid therapy and the cohort that decided against showed the following.
  - Of the eyes seen within 2 weeks of onset, there was no significant difference in the initial visual acuity and visual field defect between the untreated and treated cohorts (*P* = 0.201 for visual acuity; *P* = 0.304 for visual field defect). This is the most important fact when the end point of the study is the visual outcome.
  - There was no significant difference in gender distribution (*P* = 0.594), smoking status (*P* = 0.603), prevalence of ischemic heart disease (*P* = 0.258), peripheral vascular disease (*P* = 0.920), transient ischemic attacks/stroke (6% versus 9%; *P* = 0.097) and diabetes mellitus (27% versus 32%; *P* = 0.126).
  - The only difference was that the patients who opted for corticosteroid were slightly younger (59.2 versus 62.0 years; *P* = 0.006) and had a lower prevalence of arterial hypertension (34% versus 43%; *P* = 0.036). To determine if this influenced the visual outcome in this study, they were accounted for in the statistical analysis by including them as covariates in the logistic regression model. Statistical analysis showed that that differences in age and arterial hypertension had no significant association with the primary outcome of visual acuity (age at onset *P* = 0.817; arterial hypertension *P* = 0.589) or visual field improvement (age at onset *P* = 0.746; arterial hypertension *P* = 0.271). Thus, *the differences in age and in prevalence of arterial hypertension did not make any significant difference in the visual outcome*.
  - The visual acuity outcome in the untreated group[37] component of this study was identical to that in the untreated group in the randomized “Ischemic Optic Neuropathy Decompression Trial” study,[45] i.e., 41% versus 43%, respectively. If there were any bias, that would not have been the case.
    Thus, the concerns of the critics, with respect to validity of the results of this clinical trial not being conventionally randomized, were adequately addressed. When all these facts are put together, one can conclude that in spite of the lack of conventional randomization, this study provides scientifically valid information about the role of corticosteroid therapy in NA-AION. Moreover, the odds ratio for improvement in visual acuity from the initial visit in the steroid treated group relative to that in the natural history group was 4.45 (95% CI: 2.03, 9.75; *P* = 0.0002) at 3 months, 3.39 (95% CI: 1.62, 7.11; *P* = 0.001) at 6 months and 4.06 (95% CI: 1.92, 8.57) at 1 year. This is not at all a minor difference when the two groups were basically similar. This controversy about the role of steroid therapy in NA-AION has been discussed at length more recently elsewhere[67].
4. *Intravitreal triamcinolone acetonide*: There have recently been two small, contradictory studies on this topic. Jonas *et al*,[68] in three patients, found that it had no beneficial effect on visual acuity. Kaderli *et al*,[69] in four eyes, reported visual acuity improvement, but without any improvement in visual fields. However, the study of Kaderli *et al*,[69] has flaws which are discussed in detail elsewhere.[70] Briefly, these include the following. (a) Their study was based on only four eyes. (b) Two large natural history studies have shown spontaneous visual acuity improvement in 41–43% of eyes with NA-AION.[37,45] (c) More importantly, none of the eyes in the study by Kaderli *et al*,[69] showed improvement in visual fields and all had altitudinal visual field defects. Studies have shown that in NA-AION and A-AION, apparent visual acuity improvement without visual field improvement is due to the patient learning to fixate eccentrically, rather than being a genuine visual improvement;[37,61] In the study by Kaderli *et al*,[69] eccentric fixation may explain why the visual acuity of the patients apparently improved, while the visual fields did not.
5. *Intravitreal bevacizumab*: There is one anecdotal case report claiming reduction of optic disc edema and visual improvement after an intravitreal injection of bevacizumab (Avastin) 3 weeks after the onset of NA-AION in one eye.[71] The authors claim that bevacizumab, a vascular endothelial growth factor (VEGF) inhibitory drug, improved visual acuity in the eye by reducing optic disc edema. It is impossible to judge the effectiveness of a mode of treatment from one eye, when 41–43% of NA-AION eyes show spontaneous visual acuity improvement.[37,45] In contrast, there have recently been two reports where intravitreal bevacizumab injection actually resulted in the development of NA-AION. In one,[72] a 72-year-old woman woke up with visual loss 1 week after intravitreal injection of bevacizumab and was diagnosed to have NA-AION. In the second report,[73] a 51-year-old male “recognized” visual field defects 2 weeks after injection of intravitreal bevacizumab for choroidal neovascularization, and was diagnosed as having NA-AION. In these two cases, it could be argued that because there was a time lag of 1 and 2 weeks, respectively, between the intravitreal injection of bevacizumab and the “development” of NA-AION, the latter could not be attributed to the former. In the second report,[72] where the patient “recognized” the visual field defect, the possibility that the vision loss developed well before it was “recognized” by the patient cannot be ruled out. I have seen this in some patients with NA-AION. This delay between the intravitreal injection and development of visual loss due to NA-AION can be explained. In the clinical entity of “*Incipient NA-AION*”,[44] the NA-AION initially has asymptomatic optic disc edema, but 25% of them later on progress to classical NA-AION with median time of progression of 5.8 (interquartile 3.2–10.1) weeks from the initial diagnosis. Thus, contrary to the universal impression, the time of visual loss is not always the time of onset of NA-AION - it is only the time when the patient notices it. In these two cases,[72,73] the intravitreal injection most probably started the chain of events. It initially produced “incipient NA-AION”; however, the patients were seen by the ophthalmologist only when they developed and noticed visual loss 1–2 weeks later.
  Harmful complications following intravitreal injection of triamcinolone acetonide or anti-VEGF drugs for treatment of NA-AION
  Currently, there is a tremendous enthusiasm for the use of these drugs in all sorts of disorders in ophthalmology. As mentioned above, they have recently been advocated for the treatment of NA-AION as well. Two reports of development of NA-AION following intravitreal injection of bevacizumab are mentioned above.[72,73] Naturally the question arises: What is the mechanism of development of NA-AION following intravitreal injection of bevacizumab? The ONH circulation depends upon the perfusion pressure (*perfusion pressure = mean blood pressure minus IOP*) in its capillaries. Following intravitreal injection of anti-VEGF drugs, a short-term or severe sustained rise of IOP has been reported.
  - A short-term rise of IOP is due to increases of volume in the eyeball following intravitreal injection. After intravitreal injection of bevacizumab, Kim *et al*,[74] reported a rise of IOP to 44 mm Hg (range 4–87 mm Hg), Falkensein *et al*,[75] reported a mean IOP of 36 mm Hg, and Hollands *et al*,[76] reported an IOP of 25 mm Hg or higher at 30 minutes and one patient required a 1-week course of glaucoma medication to control the IOP. Semoun *et al*,[77] reported a case of an acute angle-closure glaucoma that occurred immediately after an intravitreal injection of 0.05 ml of bevacizumab.
  - Severe and sustained ocular hypertension has been reported following intravitreal ranibizumab for age-related macular degeneration. Bakri *et al*,[78] reported sustained long-term ocular hypertension as high as 30–50 mm Hg in four patients following 0.5 mg intravitreal ranibizumab, requiring control with topical anti-glaucoma therapy, and in two cases the addition of a systemic carbonic anhydrase inhibitor. None of the patients had a previous history of glaucoma. Kahook *et al*,[79] reported six cases of significant and sustained elevation of lOP, all requiring IOP-lowering therapy. At the annual meeting of the ARVO in May 2009, there was another report of four cases with similar persistent elevation of IOP following intravitreal injection of bevacizumab. Jalil *et al*,[80] reported a patient who was found to have an IOP of 56 mm Hg, 3 days after intravitreal injection of bevacizumab. Similarly, there are many reports showing a substantial rise in IOP a few days or weeks after intravitreal triamcinolone; by contrast, oral steroid therapy for NA-AION did not have that effect on IOP during a short-term treatment.[38] We do not have information about how common severe and sustained ocular hypertension is after intravitreal ranibizumab because the usual practice is to monitor IOP only immediately post-injection and not later on. It is possible that it may be more prevalent than reported in the literature.
    In NA-AION, the ONH already has poor and precarious circulation. Under those circumstances, even a transient high IOP, let alone prolonged, sustained high IOP, has the potential to further compromise the circulation and result in further ONH damage and visual loss. For patients who do not already have NA-AION, there is a risk of developing NA-AION with this transient or sustained high IOP if they are susceptible because of the predisposing risk factors discussed above. When treating age-related macular degeneration in those cases, on a risk/benefit ratio, it may be prudent to avoid any immediate or prolonged rise of IOP by taking precautionary measures. For persons who already have had NA-AION in one eye, the risk of the second eye developing NA-AION is much higher than for those who have never had NA-AION before. Thus, a high IOP can precipitate development of NA-AION in persons with predisposing risk factors and can worsen the visual outcome in those with NA-AION. In view of this, intravitreal injections of anti-VEGF agents or of triamcinolone have the potential to be harmful in the management of NA-AION, a factor not fully realized in the current enthusiasm for widespread use of anti-VEGF agents, particularly as a proposed treatment for NA-AION. Moreover, anti-VEGF agents rarely cause other ocular complications as well as systemic side effects, e.g., acute elevation of systemic blood pressure, cerebrovascular accidents, myocardial infarction (even some deaths have been reported).[81]
6. *Reduction of risk factors*: The usual advice given by ophthalmologists and neurologists to NA-AION patients is that nothing can be done. Having dealt with more than a thousand patients with NA-AION and having investigated various aspects of NA-AION over the years, I find that it is an inadequate response. As discussed above, NA-AION is a multifactorial disease and many risk factors contribute to it. The correct strategy is to try to evaluate and reduce as many risk factors as possible, to reduce the risk of NA-AION in the second eye or any further episode in the same eye.
  As discussed above, nocturnal arterial hypotension is a major risk factor in NA-AION patients who already have predisposing risk factors [Fig. 2]. If those drugs are taken in the evening or at bedtime, they aggravate the physiological fall of blood pressure, resulting in marked nocturnal arterial hypotension which may precipitate NA-AION in persons with predisposing risk factors [Fig. 16]. Since the 1960s, many highly potent drugs with arterial hypotensive effect have emerged to treat arterial hypertension, other cardiovascular diseases, benign prostatic hyperplasia and other diseases; those drugs are currently widely used. It may not be coincidental that the incidence of NA-AION has progressively increased since the 1960s, so that it has now become a common visually disabling disease. This strongly suggests that NA-AION may be emerging as an iatrogenic disease, stemming from the aggressive use of the very potent arterial hypotensive agents now available. In view of this, management of nocturnal arterial hypotension seems to be an important step both in the management of NA-AION and in the prevention of its development in the second eye. Therefore, I strongly recommend that when a patient is at risk of developing ocular or ONH ischemic or vascular disorders, or has the following: (a) acute NA-AION or past history of NA-AION in one eye, (b) active GCA, (c) normal-tension glaucoma, (d) occlusion or severe stenosis of internal carotid artery, (e) low central retinal artery pressure or (f) chronic optic disc edema due to any cause, the treating physician should be made aware of the potential risks of intensive arterial hypotensive therapy, particularly when giving that in the evening [Fig. 16].

### Management of PION

This depends upon the type of PION. In all cases other than surgical PION, as in AION, *the most important first step in persons aged 50 years or older is to rule out GCA always*.[52,53,57]

1. **A-PION:** Management is similar to that of A-AION discussed above. There is usually no visual improvement with systemic steroid therapy.[61] The primary objective is to prevent any further visual loss.
2. **NA-PION:** I evaluated the role of systemic steroid therapy in NA-PION.[38] This study showed that the eyes of patients treated with high-dose systemic steroid therapy during the very early stages of the disease showed significant improvement in visual acuity and visual field, compared to untreated eyes. In addition, the magnitude of visual acuity and visual field improvement was much greater in the treated group than in the untreated group. Thus, it is clear that aggressive systemic steroid therapy has a beneficial effect on visual function during the very early stages of the disease. However, spontaneous improvement in visual acuity and visual field may also occur to some extent in some eyes without steroid therapy.
  In the management of these patients, since systemic risk factors may play a part in the development of NA-PION, one should try to reduce as many risk factors as possible, to reduce the risk of second eye involvement.
3. **Surgical PION:** Basically, the management amounts to prophylactic measures to prevent its development, because once the visual loss occurs, it is usually bilateral, severe and irreversible.[13] No treatment has been found to be effective to recover or improve the lost vision. Prophylactic measures during surgery include avoiding arterial hypotension, excessive fluid replacement and hemodilution, pressure on the eyeball and orbit, and dependent position of the head, as well as shortening the duration of surgery to the minimum. There is almost a universal misconception that if a patient develops low blood pressure during surgery, the anesthetists ought to give vasopressor drugs to raise the blood pressure. This is a highly dangerous practice because vasopressor drugs raise blood pressure by causing vasoconstriction of the terminal arterioles, which results in further reduction of the blood flow in the capillary bed distal to the terminal arterioles. It is the circulation in the capillary bed that matters for tissue nutrition. Therefore, vasoconstriction of the terminal arterioles causes aggravation of ischemia of the tissue. Thus, in such cases, normal blood pressure with the use of vasopressor drugs gives a false sense of security.
  Since systemic cardiovascular risk factors may predispose a patient to a higher risk of developing surgical PION, it may be advisable to consider those factors in the decision to perform surgery.
